# An Integrative Bioinformatic Analysis of Microbiome and Transcriptome for Predicting the Risk of Colon Adenocarcinoma

**DOI:** 10.1155/2022/7994074

**Published:** 2022-01-20

**Authors:** Jieyang Yu, Cuizhen Nong, Jingjie Zhao, Lingzhang Meng, Jian Song

**Affiliations:** ^1^Baise Maternal and Child Hospital, Baise, Guangxi Zhuang Autonomous Region, China; ^2^Department of Radiation, Affiliated Hospital of Youjiang Medical University for Nationalities, Baise, Guangxi Zhuang Autonomous Region, China; ^3^Life Science and Clinical Research Center, Affiliated Hospital of Youjiang Medical University for Nationalities, Baise, Guangxi Zhuang Autonomous Region, China; ^4^Center for Systemic Inflammation Research (CSIR), School of Preclinical Medicine, Youjiang Medical University for Nationalities, Baise, Guangxi Zhuang Autonomous Region, China; ^5^Department of Radiation Oncology, Renji Hospital, School of Medicine, Shanghai Jiao Tong University, Shanghai, China

## Abstract

The abundance of gut microbiota is significantly decreased in patients with colorectal tumors compared to healthy groups. However, few studies have been conducted to correlate the differences in gut microbiota in colon cancer patients with different prognosis. In this study, we analysed the gut microbiota among patients with colon cancer and determined the microbial characteristics of COAD and divided the overall survival of COAD data into the high- and low-risk groups. In addition, we established a microbiome-related gene map and determined the association between microbial features and immune cell infiltration in COAD. In comparison with the low-risk group, the high risk group of COAD samples exhibited a decreased proportion of activated CD4 T cells as well as an increased proportion of M2 macrophages. The current data suggested that different gut flora backgrounds lead to different gene expression profiles, which in turn affect immune cell typing and colorectal tumor prognosis.

## 1. Introduction

Colon cancer is one of the leading causes of cancer deaths worldwide [1], and there are many studies on the molecular mechanisms of colon carcinogenesis, among which the regulation of the intestinal microecosystem that determines the expression of oncogenes is a hot topic [2]. Fusobacterium nucleatum and anaerobic streptococci can promote the occurrence, development, and treatment of colorectal tumors [3, 4]; probiotics such as lactobacilli can inhibit the occurrence and development of colorectal tumors and can improve postoperative indicators and reduce complications in patients with colorectal cancer [5]. Therefore, it is important to genotype the gut microbiota profile before treating patients with colon cancer, which requires an understanding of the correlation between the gut microbiota profile and the overall risk of colon cancer.

It has been known that both innate and adaptive immunities are involved in the development of tumors and gut microbiota play a key role in regulating the intestinal immunity [3, 6]. The altered immunity predisposes the host to acquire adenomatous or carcinogenic changes or impact the prognosis of the colorectal cancer patients. For example, Fusobacterium nucleatum induces mucin secretion and inflammatory cytokines during contact or invasion of colonic cells and also inhibits the immune process against tumors and suppresses the activity of natural killer cells, thus promoting the development of colorectal cancer [7]. However, few comprehensive studies have been conducted to correlate the differences in gut microbiota in colon cancer patients with their immune cell profiles and the prognosis of patients.

This study analysed the composition and differences in the gut microbiota of colon cancer patients. Significant differences in the gut microbiota of colon cancer patients were found at the family levels. We used the corresponding gut microbiota to study the prognostic risk and grouping of colon cancer patients. And differential gene expression profiles associated with them were identified.

## 2. Materials and Methods

### 2.1. Collection and Processing of the COAD RNAseq Dataset

The RNA sequencing dataset and the clinically related data for COAD originated from the TCGA database (https://portal.gdc.carcinoma.gov/), which consists of 410 samples. The raw gene expression dataset was processed. Probe IDs received the annotation toward the gene from the corresponding platform annotation profile of the GDC website, and the raw matrix data received the quantile normalization and log2 conversion. Samples with missing data were excluded.

### 2.2. Building a Microbial Signature

The association between COAD microbiome and overall survival time in TCGA cases was studied. Univariate Cox regression analysis was carried out for identifying the genes associated with survival (*p* value < 0.05). Subsequently, the significance of candidate genes was selected using variable importance in a randomized survival forest (RSF) algorithm. A risk score model with the selected microbial signature was built using multivariate Cox regression approaches. In addition, the Kaplan-Meier test was employed for a number of gene features, and *p* values (log) were determined. Receiver operating characteristic (ROC) analysis was performed for 3- and 5-year overall survival rates, and area under the curves (AUCs) were determined for assessing the specificity and sensitivity of the microbial signature.

### 2.3. Microbiome Analysis

Based on the operational taxonomic unit (OTU) results generated by sample sequencing, the phyloseq R package was used to calculate the alpha diversity distance matrices. Microbiome analysis was otherwise done using microbiomeanalyst.ca website. Alpha diversity refers to the diversity of species in a given region or ecosystem and is commonly measured by the Shannon's and Simpson's indices. Shannon's index is correlated with the diversity, while Simpson's index is inversely proportional to the diversity. Beta diversity analysis is a comparative analysis between groups of species diversity among different ecosystems or microbial communities to obtain similarities or differences in community composition among different grouped samples.

### 2.4. Statistical Methods

Statistics investigations were carried out with R software (version 3.6.0). Kaplan-Meier tests and ROC analysis were performed as we described previously [8, 9]. In brief, we utilized the “survivor” and “survROC” software packages in both analyses [10]. Optimal cut-off data points were calculated using the “survminer” package [11]. Single-variate and multivariate Cox regression correlations were used to assess the prognosis-correlated factors of interest. Hazard ratios and 95% confidence intervals were presented for all the prognosis-correlated factors.

Analysis of differences between groups was performed using GraphPad Prism 8.0 software. Measures were shown as the mean ± standard deviation. Student *t*-tests were used for comparisons between two groups if they met a normal distribution and had a homogeneous variance.

## 3. Results

### 3.1. Building a Microbial Signature

**Figure 1:**
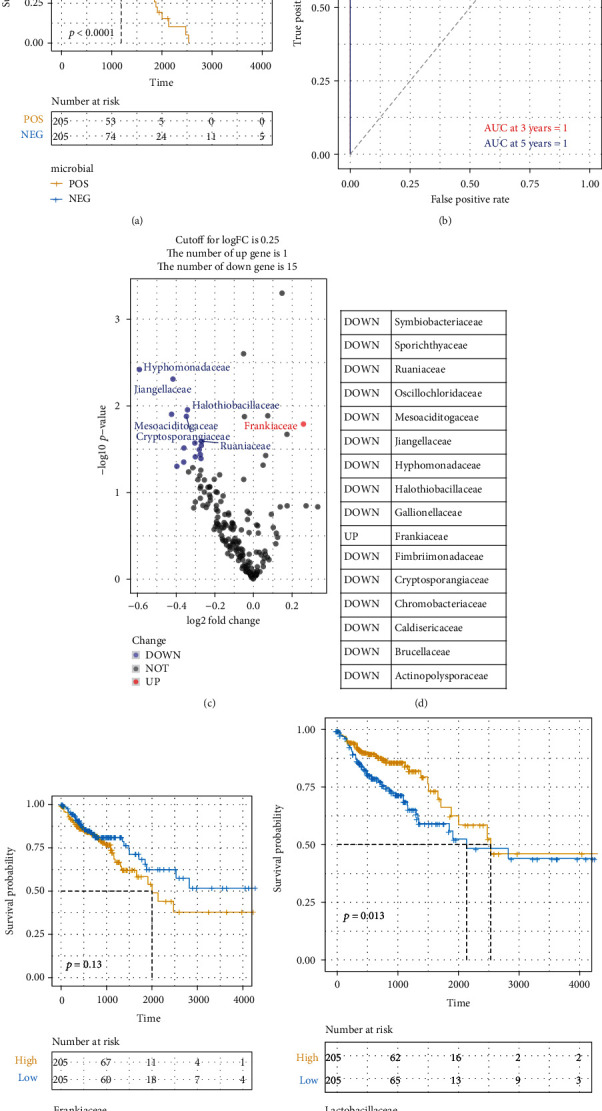
Identification of COAD prognosis correlated microbiome. (a) Kaplan-Meier (KM) analysis of the risk group that was defined with prognosis correlated microbiome in the TCGA dataset for COAD. (b) Three- and five-year ROC survival curves of the risk groups for COAD TCGA dataset. (c) A volcano plot of the differential microbials in the two risk groups of COAD. (d) List of the top differential microbials. Kaplan-Meier (KM) survival analysis of (e) Frankiaceae and (f) Lactobacillaceae in the COAD patients.

COAD tumor microbiomes were obtained from the pan cancer microbiome of cBioportal website and then integrated with their respective clinical data. To screen for the crucial survival-related factors, the microbial from COAD tumor were analysed using multivariate Cox regression for the TCGA dataset, and the risk scoring system was then built using these 180 microbials with multivariate Cox analysis using the TCGA clinical dataset (Table 1). In accordance with the formula, a risk score was calculated for the respective cases. COAD tumor in the TCGA dataset was then divided into the high-risk and low-risk cohorts with the optimal cut-off data for the risk score. Kaplan-Meier curves showed that the high-risk group survived for shorter periods in comparison with those patients in the low-risk cohort (Figure 1(a)). ROC curve analysis of the COAD cases was plotted, and this showed an AUC of 1 for 3- and 5-year survivals (Figure 1(b)). Subsequently, the microbials were screened based on with the best cut-off data, and 15 were significantly downregulated and Frankiaceae was upregulated (Figures 1(c) and 1(d)). Kaplan-Meier curves also confirmed that Frankiaceae is associated with the high risk and Lactobacillaceae is classified as the beneficial factor (Figures 1(e) and 1(f)).

### 3.2. Microbiome Analysis of the High- and Low-Risk Groups

**Figure 2:**
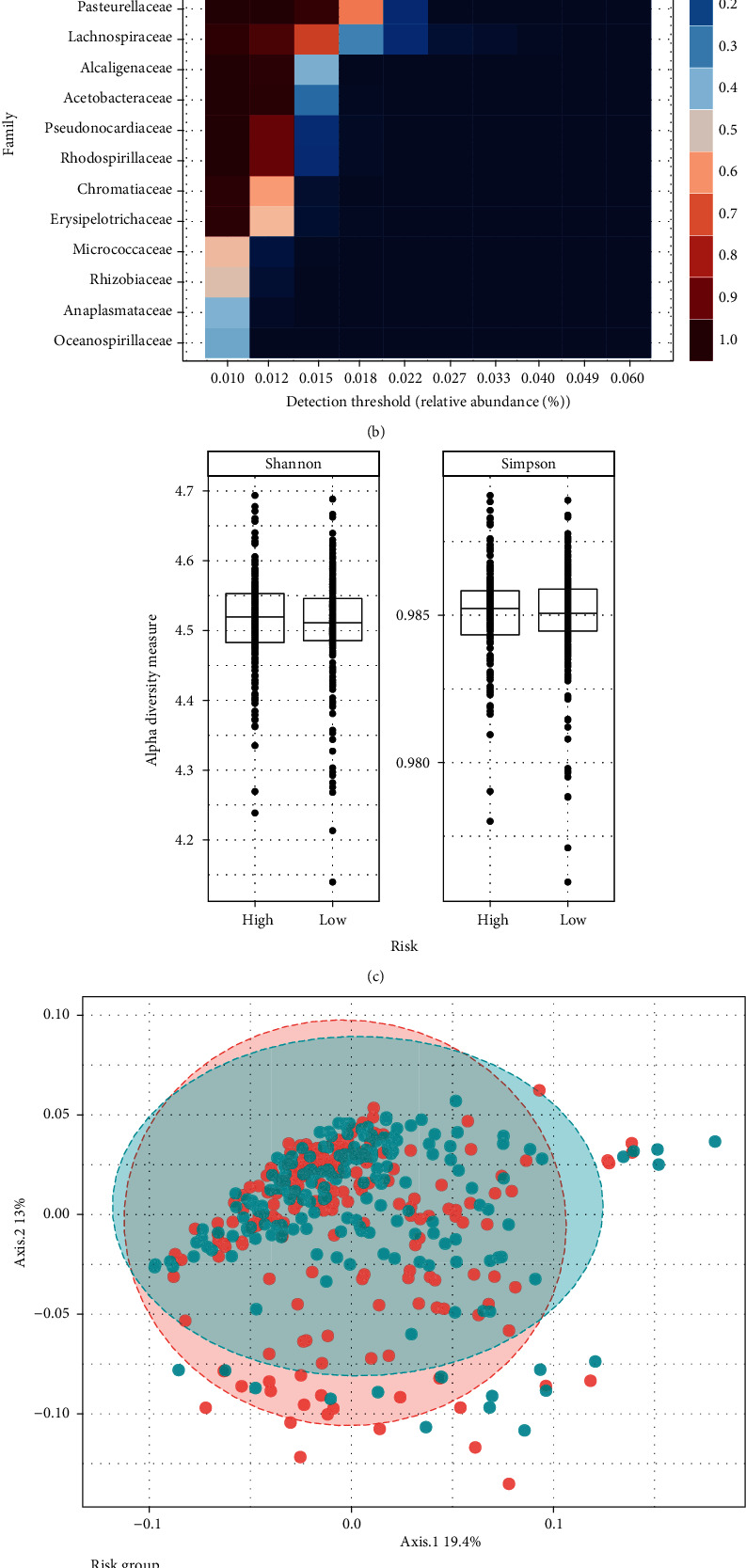
Microbiome analysis of the high- and low-risk groups of COAD. (a) Composition of the gut microbiota at the phylum level in patients with high and low risk. (b) Core microbiome analysis of family level. (c) Alpha diversity analysis of samples in the high and low risk dataset. (d) Two-dimensional scatter plot of nonmetric multidimensional scale analysis of gut microbiota family gate levels in COAD cancer patients. (e) A random forest “classification” approach was used to find key bacteria associated with groupings.

The analysis of the community composition both COAD groups showed the gut microbiota in patients with colon and rectal cancer at the Phylum level (Figures 2(a) and 2(b)). Based on the genus level, alpha diversity analysis of samples from the high- and low-risk groups showed that the difference in Shannon's index was not statistically significant; Simpson's index was also not statistically significant (Figure 2(c)). NMDS analysis showed that at the family level, the difference in bacterial gut microbiota community between the two groups was significant (Figure 2(d)), suggesting a significant difference in the composition of gut microbiota between high- and low-risk patients. A random forest “classification” approach was used to find key bacteria associated with groupings (Figure 2(e)).

### 3.3. DEG Identification

**Figure 3:**
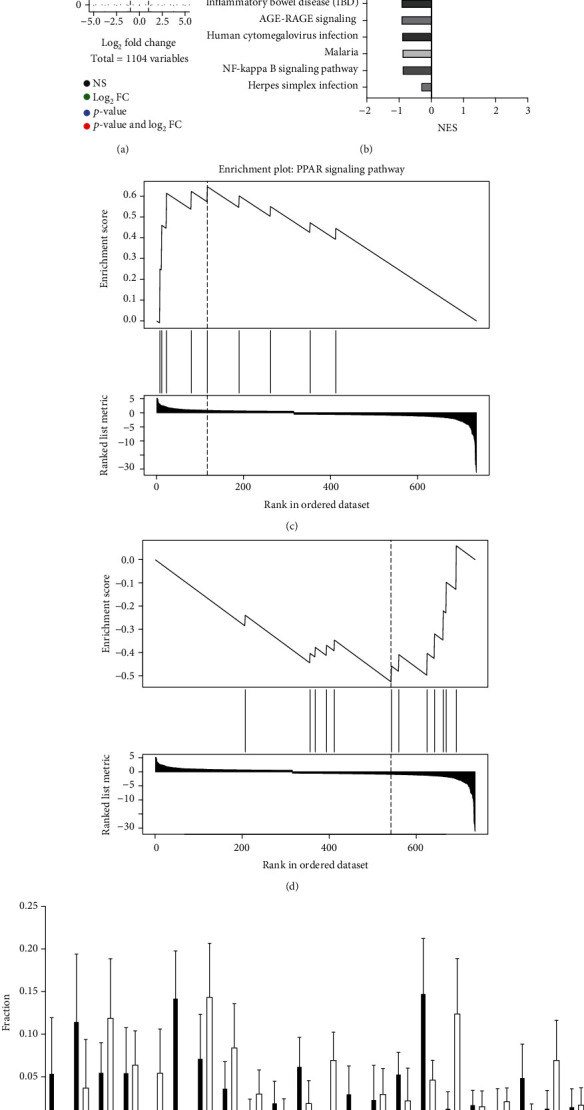
Profiling the gene expression of the high-risk microbiome-related genes. (a) A volcano plot of the DEGs between high- and low-risk groups of COAD samples. (b) Bar graphs showing the enriched KEGG pathways of the risk DEGs. GSEA plots showed the upregulated (c) PPAR signaling and the downregulated (d) IL17 signaling. (e) Bar graphs showing the CIBERSORT estimated infiltration of immune cell subsets into the COAD high- and low-risk samples.

COAD cases in the TCGA dataset were categorized according to high- or low-risk score. To screen for the crucial survival-related factors, the DEGs from the two risk groups were analysed with Wilcoxon's test and were enriched for the KEGG pathway analyses (Figures 3(a) and 3(b)). GSEA KEGG pathway analyses were done using webgestalt.org website [8]. Of those pathways, PPAR signaling was upregulated and the IL17 signaling was decreased in the high risk group (Figures 3(c) and 3(d)). Interestingly, IL1beta and IL23 were downregulated, which refers to a decreased IBD signaling (data not shown). These function data are correlated with the estimated infiltration of immune cell subsets into the COAD tumor samples, which is obtained using the CIBERSORT deconvolution of COAD bulk-seq data (Figure 3(e)).

### 3.4. Constructing a Microbial-Related Gene Signature

**Figure 4:**
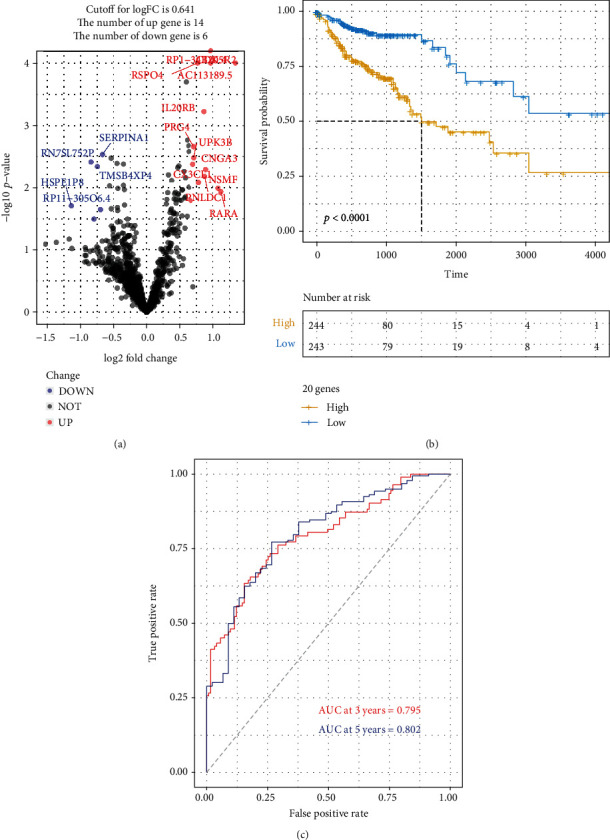
Constructing a microbiome related prognosis gene signature for COAD. (a) A volcano plot showing the significant genes obtained from Cox regression analysis of survival-related DEGs in the high- and low-risk groups of COAD samples. (b) KM investigation of the risk model for the significant gene signatures. (c) Three- and five-year ROC curves of COAD TCGA dataset for the gene signatures.

The DEGs of the COAD high- or low-risk groups were analyzed using single-variate Cox regression for the TCGA dataset, and a total of 2 genes were identified to be significantly correlated to survival in these patients (*p* < 0.05) (Figure 4(a)). The risk scoring system was then built using these 20 genes with multivariate Cox analysis using the TCGA dataset. Kaplan-Meier curves showed that the high-expressing group survived for fewer periods in comparison with those patients in the low-risk cohort (Figure 4(b)). To estimate the predictive power of genetic characteristics, ROC curve analysis of the COAD cases were plotted and this showed an AUC of 0.795 for 3 year-survival and 0.802 for 5 year-survival (Figure 4(c)). This provides a potential microbial-related gene signature for the assessment of COAD patients before their further treatment.

## 4. Discussion

After human birth, a large number of microorganisms start to colonize and reproduce in the intestine, inducing the formation of the body's immune system and playing an important role in body homeostasis [12]. In addition to gastrointestinal-related diseases, dysbiosis of gut microbiota also plays a contributing role in the pathogenesis of Parkinson's disease and Alzheimer's disease, and microbiomics has become a major research hotspot at present [13, 14]. Previous studies have demonstrated that the abundance of gut microbiota is significantly decreased in patients with colorectal tumors compared to healthy groups [15]. However, few studies have been conducted to correlate the differences in gut microbiota in colon cancer patients with different prognoses. In this study, we analysed the composition and differences of gut microbiota among patients with colon cancer.

Using a Cox proportional risk model, we determined the microbial characteristics of COAD and divided the overall survival of COAD data into two risk groups, with high-risk cases showing a poorer prognosis. In addition, we established a microbiome-related gene map and determined the association between microbial features and immune cell infiltration in COAD using the TCGA dataset. The high-risk group of COAD samples exhibited a decreased proportion of activated CD4 T cells as well as an increased proportion of M2 macrophages, indicating the impact of high risk microbials on the activation and differentiation of macrophage and T cells (Figure 3(e)). However, a further study would be expected to investigate the underlying mechanism.

The composition and function of intestinal commensal bacteria vary among colon cancers with different prognoses, as do the interactions between hosts and microorganisms. The entire intestine is a vast mutually beneficial ecosystem. By dividing the intestinal commensal gut microbiota into additional subgroups, we can gain a clearer understanding of the molecular mechanisms of action of the different gut microbiota. Dysbiosis and translocation are strongly associated with colorectal cancer, and microbiome analyses are expected to be very valuable noninvasive biomarkers for colorectal cancer diagnosis. In addition, gut microbiota are potential therapeutic targets to inhibit the proliferation and metastasis of colorectal cancer.

It is well known that the successful application of immune checkpoint blockade is attributed to the ability of the antitumor immune response, which largely depends on T cell activation. However, tumor-infiltrated cells often exhibit T-cell exhaustion. In this study, we explored the tumor immune environment using the information of microbiomics from COAD patients and constructed a prognostic signature, which may facilitate to screen patients for immunotherapy. However, our study was retrospective. In the future, the researchers need more prospective studies to further apply and validate our findings.

## Figures and Tables

**Table 1:** Top gut microbial correlating with high or lower risk of colon cancer mortality.

Gene	Coefficie nt	P value	Gene	Coefficient	P value
Piscirickettsiaceae	247.19	P < 0.001	Mesoaciditogaceae	-140.792	P < 0.001
Syntrophaceae	114.643	P < 0.001	Hyphomonadaceae	-107.232	P < 0.001
Listeriaceae	87.535	P < 0.001	Bacillaceae	-85.157	P < 0.001
Aquificaceae	86.796	P < 0.001	Thermoanaerobacterales_Family_IV._Incertae_Sedis	-72.364	P < 0.001
Comamonadaceae	79.195	P < 0.001	Peptococcaceae	-61.483	P < 0.001
Ignavibacteriaceae	62.405	P < 0.001	Alcanivoracaceae	-59.424	P < 0.001
Mariprofundaceae	54.515	P < 0.001	Actinomycetaceae	-56.622	P < 0.001
Chromobacteriaceae	41.484	P < 0.001	Syntrophorhabdaceae	-56.583	P < 0.001
Limnochordaceae	40.748	P < 0.001	Rickettsiaceae	-54.024	P < 0.001
Acidothermaceae	39.392	P < 0.001	Ruminococcaceae	-53.921	P < 0.001
Phyllobacteriaceae	36.895	P < 0.001	Rhodospirillaceae	-48.748	P < 0.001
Symbiobacteriaceae	35.305	P < 0.001	Alicyclobacillaceae	-43.438	P < 0.001
Bacteroidaceae	35.118	P < 0.001	Oscillochloridaceae	-41.465	P < 0.001
Salinisphaeraceae	35.031	P < 0.001	Gallionellaceae	-39.995	P < 0.001
Chitinivibrionaceae	34.137	P < 0.001	Methylothermaceae	-37.641	P < 0.001
Marinilabiliaceae	33.292	P < 0.001	Mycobacteriaceae	-36.452	P < 0.001
Frankiaceae	33.195	P < 0.001	Caldisericaceae	-36.349	P < 0.001
Marinifilaceae	32.807	P < 0.001	Sporichthyaceae	-35.514	P < 0.001
Oleiphilaceae	32.446	P < 0.001	Cryptosporangiaceae	-33.537	P < 0.001
Halobacteriovoraceae	31.806	P < 0.001	Burkholderiaceae	-33.496	P < 0.001
Puniceicoccaceae	30.899	P < 0.001	Fimbriimonadaceae	-32.953	P < 0.001
Magnetococcaceae	28.051	P < 0.001	Kordiimonadaceae	-31.766	P < 0.001
Nannocystaceae	26.418	P < 0.001	Saccharospirillaceae	-31.468	P < 0.001
Porticoccaceae	25.824	P < 0.001	Jiangellaceae	-29.333	P < 0.001
Nakamurellaceae	24.696	P < 0.001	Promicromonosporaceae	-28.741	P < 0.001
Eubacteriaceae	23.719	P < 0.001	Legionellaceae	-26.135	P < 0.001
Rubrobacteraceae	23.458	P < 0.001	Cellvibrionaceae	-21.792	P < 0.001
Ferrimonadaceae	22.347	P < 0.001	Gordoniaceae	-21.702	P < 0.001

## Data Availability

The datasets presented in this study can be found in online repositories. The names of the repository/repositories and accession number(s) can be found in the article.
